# Molecular fingerprinting of multidrug-resistant Salmonella enterica serotype Typhi.

**DOI:** 10.3201/eid0402.980223

**Published:** 1998

**Authors:** M D Hampton, L R Ward, B Rowe, E J Threlfall

**Affiliations:** Central Public Health Laboratory, London, United Kingdom.

## Abstract

For epidemiologic investigations, the primary subdivision of Salmonella Typhi is vi-phage typing; 106 Vi-phage types are defined. For multidrug-resistant strains the most common types have been M1 (Pakistan) and E1 (India, Pakistan, Bangladesh, and the Arabian Gulf); a strain untypable with the Vi phages has been responsible for a major epidemic in Tajikistan. Most often, isolates from the Indian subcontinent have been resistant to ampicillin, chloramphenicol, streptomycin, sulfonamides, tetracyclines, and trimethoprim; but in the 1997 Tajikistan outbreak, the epidemic strain was also resistant to ciprofloxacin. For multidrug-resistant strains, subdivision within phage type can be achieved by plasmid profile typing and pulsed-field gel electrophoresis.


### Molecular Fingerprinting of Multidrug-Resistant *Salmonella enterica* Serotype Typhi

**M. D. Hampton, L. R. Ward, B. Rowe, and E. J. Threlfall**
Central Public Health Laboratory, London, United Kingdom

For epidemiologic investigations, the primary subdivision of *Salmonella* Typhi is vi-phage typing; 106 Vi-phage types are defined. For multidrug-resistant strains the most common types have been M1 (Pakistan) and E1 (India, Pakistan, Bangladesh, and the Arabian Gulf); a strain untypable with the Vi phages has been responsible for a major epidemic in Tajikistan. Most often, isolates from the Indian subcontinent have been resistant to ampicillin, chloramphenicol, streptomycin, sulfonamides, tetracyclines, and trimethoprim; but in the 1997 Tajikistan outbreak, the epidemic strain was also resistant to ciprofloxacin. For multidrug-resistant strains, subdivision within phage type can be achieved by plasmid profile typing and pulsed-field gel electrophoresis.

In the United Kingdom, the incidence of multidrug-resistant (MDR) *Salmonella* Typhi isolates has increased from 1.5% in 1989 to 34% in 1995 (1). The most common MDR Vi-phage types have been M1 and E1, isolated from patients who recently returned from Southeast Asia or the Indian subcontinent (1). Although the increasing incidence of MDR isolates of Vi-phage types El and M1 has been particularly noteworthy, outbreaks caused by drug-sensitive strains of these Vi-phage types have continued to be identified. For example, all four patients in a July 1997 outbreak had recently traveled to the Dominican Republic; the causative strain was drug sensitive and belonged to Vi-phage type E1 (2).

Discrimination within phage type is desirable for studying the epidemiology of MDR strains of *S.* Typhi and for determining their phylogenetic relationships with established drug-sensitive types. Insertion sequence *200* profiling can differentiate between drug-sensitive and drug-resistant strains of Vi-phage types M1 and E1 but is unsuitable for subdivision within these phage types (3).

We used plasmid profile typing and pulsed-field gel electrophoresis (PFGE) for the molecular characterization and subdivision of isolates of *S.* Typhi of Vi-phage types E1, M1, and untypable Vi strain (UVS) from outbreaks and sporadic cases. We selected a panel of 18 strains comprising the type strains of *S.* Typhi Vi-phage types E1 and M1, as well as drug-sensitive and drug-resistant isolates of these phage types from patients infected in several countries of the Indian subcontinent. We also included UVS and Vi-phage type E1 isolates from the 1997 epidemic in Tajikistan and an isolate of Vi-phage type E1 from an infection acquired in the Dominican Republic in 1997 (Table). The type strains of Vi-phage types E1 and M1 were isolated in Russia and Canada in 1918 (E1) and 1939 (M1), which demonstrates the subsequent global dissemination of these phage types over a prolonged period.

All drug-sensitive isolates of Vi-phage types E1 and M1 were plasmid-free, whereas MDR isolates were characterized by a plasmid of approximately 120 megadaltons (MDa). The two UVS isolates also had a 120-MDa plasmid and an additional plasmid of 80 MDa. Conjugation and incompatibility testing showed that the 120-MDa plasmids encoded resistance to ampicillin, chloramphenicol, streptomycin, sulphonamides, tetracyclines, and trimethoprim and belonged to incompatibility group $H_1$. In contrast, the 80-MDa plasmids in the two UVS isolates from Tajikistan did not code for drug resistance and were not further characterized by incompatibility

Address for correspondence: E. J. Threlfall, WHO Collaborating Centre for Phage Typing and Resistance in Enterobacteria, Laboratory of Enteric Pathogens, Central Public Health Laboratory, 61 Colindale Avenue, London NW9 5HT, United Kingdom; fax: 44-181-905-9929; e-mail: jthrelfall@phls.co.uk.

Table. Drug-resistant and drug-sensitive isolates of *Salmonella* Typhi phage types E1, M1, and UVS

| Laboratory accession number | Phage type | Country of origin | Year of isolation | Antibiogram[a] | Mol wt of plasmid DNA | | Pulsed-field profile |
|---|---|---|---|---|---|---|---|
| E1 | E1 | Russia | 1918 | $ | -[b] | | X1 |
| P3047990 | E1 | India | 1993 | $ | - | | X2 |
| P2967750 | E1 | India | 1992 | ACSSuTTm | 120 | | X2a |
| P3639160 | E1 | Pakistan | 1994 | ACSSuTTm | 120 | | X2a |
| P3640980 | E1 | Pakistan | 1994 | ACSSuTTm | 120 | | X2a |
| P4632360 | E1 | Bangladesh | 1997 | ACSSuTTm | 120 | | X2a |
| P4663970 | E1 | Bangladesh | 1997 | ACSSuTTm | 120 | | X2a |
| P4405140 | UVS | Tajikistan | 1997 | ACSSuTTmCp | 120 | 80 | X2a |
| P4405200 | UVS | Tajikistan | 1997 | ACSSuTTmCp | 120 | 80 | X2a |
| P4466680 | E1 | India | 1997 | ACSSuTTm | 120 | | X3 |
| P2951290 | E1 | Bangladesh | 1992 | ACSSuTTm | 120 | | X4 |
| P3166750 | E1 | Bangladesh | 1993 | ACSSuTTm | 120 | | X4 |
| P4219910 | E1 | Pakistan | 1997 | ACSSuTTmCp | 120 | | X4 |
| P4405160 | E1 | Tajikistan | 1997 | ACSSuTTm | 120 | | X5 |
| P4543210 | E1 | Dominican Republic | 1997 | $ | - | | X6 |
| M1 | M1 | Canada | 1939 | $ | - | | X7 |
| P3112100 | M1 | Pakistan | 1992 | $ | - | | X8 |
| P3044890 | M1 | Pakistan | 1992 | ACSSuTTm | 120 | | X8a |

[a]A, ampicillin; C, chloramphenicol; S, streptomycin; Su, sulphonamides; T, tetracyclines; Tm, trimethoprim; Cp, ciprofloxacin; $, drug-sensitive.
[b]Plasmid DNA not detected.

grouping. In isolates of Vi-phage type E1 and UVS with additional resistance to ciprofloxacin, resistance to this antimicrobial was chromosomally encoded.

PFGE following digestion of genomic DNA with *Xba* I showed 19 to 23 fragments per strain, with 13 fragments common to each and 1 to 12 band differences between patterns. Several differences were identified, both between and within phage types (Figure 1). For Vi-phage type E1, six pulsed-field profiles (PFPs) (*S.* Typhi PFP *Stp* X1 through PFP *Stp* X6) and one subtype (PFP *Stp* X2a) were identified and defined. For PFPs *Stp* X1, X2, X5, and X6, subdivision was based on the numbers of fragments (from 150 kb to >500 kb) and for PFPs X3 and X4, subdivision was based on the number and size of fragments in the 20-kb to 50-kb range, with PFP X3 also lacking a 300-kp band present in PFP X4. The PFPs of the two UVS isolates from Tajikistan were indistinguishable from PFP X2a. For Vi-phage type M1, two PFP types (PFPs X7 to X8) and one subtype (PFP X8a) were identified. PFPs X7 and X8 were designated as separate types on the basis of the numbers of fragments in the 250-kb to 500-kb range. PFP X2a could be distinguished from PFP X2, and PFP X8a could be distinguished from PFP X8 by the presence of three additional fragments of approximately 50 kb, 30 kb, and 25 kb. As these three fragments were observed in isolates of PFPs X2a, X3, X4, X5, and X8a, all of which had incompatibility group $H_1$ plasmids but were not identified in isolates of PFPs X1, X2, X6, X7, and X8 (none of which possessed $H_1$ plasmids), it is likely that these three fragments are plasmid-derived. These plasmid-containing derivatives of PFPs X2 and X8 have been designated PFPs X2a and X8a, respectively.

A dendogram (Figure 2**)** analyzed by pairwise fragment comparison using the Dice coefficient and by data clustering using the unweighted pair group arithmetic averaging method (UPGMA) clearly illustrated the relationships described above. The Dice coefficient values, excluding plasmid DNA, were from 66.7% to 97.6%. The PFPs of Vi-phage type E1 and UVS and Vi-phage type M1 clustered as two distinct groups with the branch-point for the E1 and UVS PFPs X1-X6 cluster at approximately 80.5% and the M1 PFPs X7-X8 cluster at approximately 86%. Within Vi-phage type E1, PFPs X1 and X6 (isolates from Russia and the Dominican Republic) are distinct from the closely related PFP X2-X5 cluster (the Indian subcontinent and Tajikastan), which

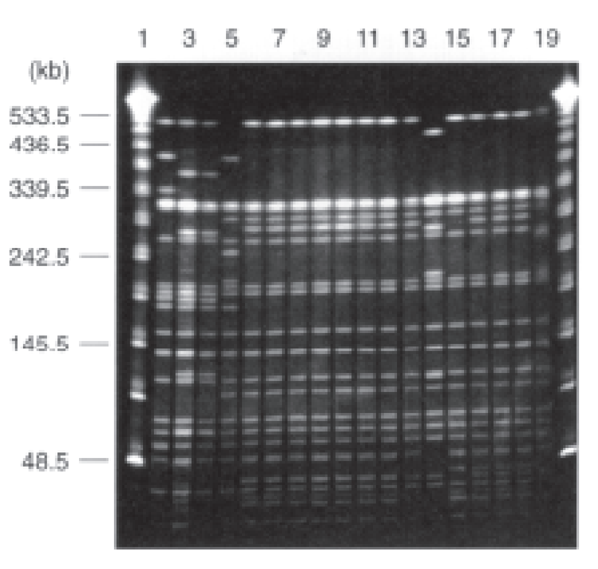

Figure 1. Pulsed-field gel electrophoresis profiles of *Xba* I-digested genomic DNA from drug-resistant and drug-sensitive isolates of *Salmonella* Typhi Vi-phage types E1, M1, and UVS. Tracks 1-20 contained 1 and 20, lambda 48.5-kb ladder (Sigma); 2, M1 type strain (*S*. Typhi PFP *Stp* X7); 3, P3044890 (PFP X8a); 4, P3112100 (PFP X8); 5, E1 type strain (PFP X1); 6, P3640980 (PFP X2a); 7, P3639160 (PFP X2a); 8, P2967750 (PFP X2a); 9, P4405140 (PFP X2a); 10, P4405200 (PFP X2a); 11, P4632360 (PFP X2a); 12, P4663970 (PFP X2a); 13, P3047990 (PFP X2); 14, P4543210 (PFP X6); 15, P4466680 (PFPX3); 16, 3166750 (PFP X4); 17, P2951290 (PFP X4); 18, P4219910 (PFP X4); and 19, P4405160 (PFP X5). PFGE conditions: 180V (5.4 V/cm); 45h; Ramp, 0.5 - 45s.

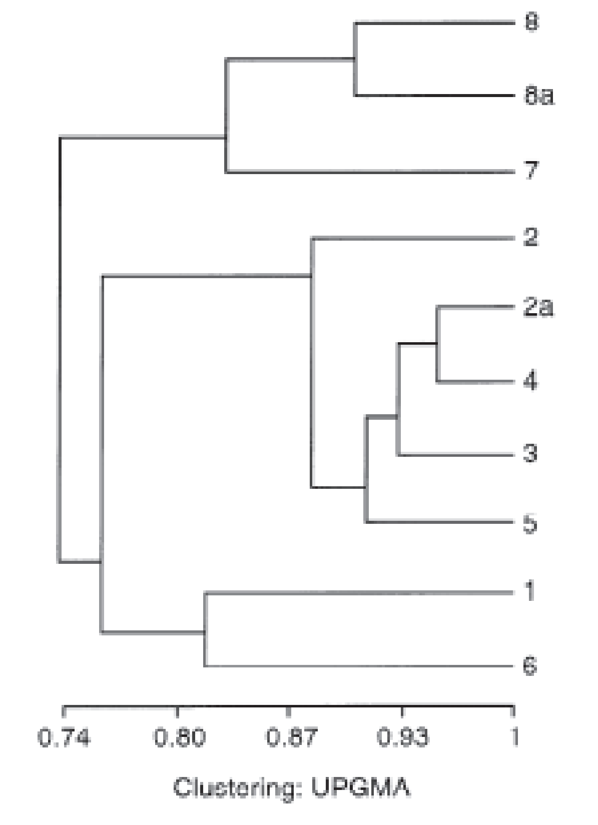

Figure 2. Dendogram of *Salmonella* Typhi pulsed-field profiles *Stp* X1 -X8a. Genetic similarity was calculated by the Dice coefficient (*S*) and clustered by unweighted pair group arithmetic averaging method.

suggests a recent evolutionary divergence in Asia within this universally common phage type.

These results demonstrate that PFGE, coupled with plasmid profile analysis, is a useful method for discriminating MDR isolates of *S*. Typhi Vi-phage type E1. The results also suggest that isolates of Vi-phage type E1 with the X2a pulsed-field profile have been derived within the Indian subcontinent from a drug-sensitive strain of Vi-phage type E1 already endemic in this area. This drug-sensitive progenitor strain is genotypically distinct from the type strain of Vi-phage type E1, isolated in Russia in 1918 and more recently in the Dominican Republic. In turn, isolates of Vi-phage type E1 of PFPs X3-X5 are closely related to PFP X2 and could have been derived from PFP X2 in the course of its recent epidemic history. The two UVS isolates from Tajikistan had a PFP indistinguishable from PFP X2a but differed by plasmid profile and antibiogram and by at least three bands from that of the MDR Vi-phage type E1 isolate from Tajikistan (PFP X5). This suggests that despite the recent observation that MDR strains of Vi-phage type E1 and UVS are in circulation in Tajikistan (4), the epidemic ciprofloxacin-resistant UVS strain is more likely to have originated in the Indian subcontinent than in Tajikistan. Likewise, the drug-sensitive and drug-resistant isolates of Vi-phage type M1 from Pakistan are genotypically distinct from the type strain of Vi-phage type M1 isolated in Canada in 1939. Again, this suggests that MDR strains of Vi-phage type M1 have been derived in Pakistan from a drug-sensitive progenitor strain of Vi-phage type M1 already endemic in that country.

A previous PFGE-based study of *S*. Typhi from Southeast Asia demonstrated that multiple genetic variants associated with sporadic cases and occasional outbreaks are simultaneously present in that area (5). The study also demonstrated considerable genetic diversity between strains, suggesting considerable movement of these strains within endemic-disease regions. However, the study did not use Vi-phage typing for the preliminary identification and subdivision of isolates. In our study, we combined Vi-phage typing with plasmid analysis and PFGE to provide a method of subdividing MDR isolates of Vi-phage types El and M1 endemic in India, Pakistan, and Bangladesh; we also compared the resulting patterns with those from historic isolates and isolates from recent outbreaks of MDR *S*. Typhi in Tajikistan. Although further work is needed to establish the applicability of combining phage typing with plasmid typing and PFGE for studying the epidemiology of MDR *S*. Typhi, our studies demonstrate that subdivision within the most common phage types can be achieved and may be useful both for investigating outbreaks and for providing information about the evolutionary history of MDR epidemic strains.

### Acknowledgments

We thank Drs. D. Murdoch and N. Banatvala of MERLIN for providing the isolates of *S*. Typhi from the 1997 epidemic in Tajikistan.

M.D. Hampton is a biomedical scientist.

### References

1. Rowe B, Ward LR, Threlfall EJ. Multiresistant *Salmonella typhi*—a world-wide epidemic. Clin Infect Dis 1997;24:S106-9.
2. Typhoid fever associated with travel to the Dominican Republic. Commun Dis Rep CDR Weekly 1997;7:299-302.
3. Threlfall EJ, Torre E, Ward LR, Rowe B, Gibert I. Insertion sequence IS*200* can differentiate drug-resistant and drug-sensitive *Salmonella typhi* of Vi-phage types E1 and M1. J Med Microbiol 1993;39:454-8.
4. Murdoch DA, Banatvala N, Bone A, Shoismatulloev BI, Ward LR, Threlfall EJ. Epidemic ciprofloxacin-resistant *Salmonella typhi* in Tajikistan. Lancet 1998;351:339.
5. Thong K-L, Puthucheary S, Yassim RM, Sudarmono P, Padmidewi M, Soewandojo E, et al. Analysis of *Salmonella typhi* isolates from southeast Asia by pulsed-field gel electrophoresis. J Clin Microbiol 1995;33:1938-41.
